# The effects of acceptance and commitment therapy on eating behavior and diet delivered through face-to-face contact and a mobile app: a randomized controlled trial

**DOI:** 10.1186/s12966-018-0654-8

**Published:** 2018-02-27

**Authors:** Elina Järvelä-Reijonen, Leila Karhunen, Essi Sairanen, Joona Muotka, Sanni Lindroos, Jaana Laitinen, Sampsa Puttonen, Katri Peuhkuri, Maarit Hallikainen, Jussi Pihlajamäki, Riitta Korpela, Miikka Ermes, Raimo Lappalainen, Marjukka Kolehmainen

**Affiliations:** 1 Institute of Public Health and Clinical Nutrition, Clinical Nutrition, University of Eastern Finland, P.O. Box 1627, FI-70211 Kuopio, Finland; 2 Institute of Clinical Medicine and Clinical Nutrition, Kuopio University Hospital, P.O. Box 100, FI-70029 KYS Kuopio, Finland; 3 Department of Psychology, University of Jyväskylä, P.O. Box 35, FI-40014 University of Jyväskylä Jyväskylä, Finland; 4 Department of Psychology, Karlstad University, SE-651 88 Karlstad, Sweden; 5 Medical Faculty, Pharmacology, Medical Nutrition Physiology, University of Helsinki, P.O. Box 63, FI-00014 University of Helsinki Helsinki, Finland; 6 Finnish Institute of Occupational Health, P.O. Box 40, FI-00251 Helsinki, Finland; 7 VTT Technical Research Centre of Finland, P.O. Box 1300, FI-33101 Tampere, Finland

**Keywords:** ACT, Behavior change, Mindfulness, Mindful eating, Intuitive eating, Dietary intake, Regulation of eating behavior, Overweight, Obesity, mHealth

## Abstract

**Background:**

Internal motivation and good psychological capabilities are important factors in successful eating-related behavior change. Thus, we investigated whether general acceptance and commitment therapy (ACT) affects reported eating behavior and diet quality and whether baseline perceived stress moderates the intervention effects.

**Methods:**

Secondary analysis of unblinded randomized controlled trial in three Finnish cities. Working-aged adults with psychological distress and overweight or obesity in three parallel groups: (1) ACT-based Face-to-face (*n* = 70; six group sessions led by a psychologist), (2) ACT-based Mobile (*n* = 78; one group session and mobile app), and (3) Control (*n* = 71; only the measurements). At baseline, the participants’ (*n* = 219, 85% females) mean body mass index was 31.3 kg/m^2^ (SD = 2.9), and mean age was 49.5 years (SD = 7.4). The measurements conducted before the 8-week intervention period (baseline), 10 weeks after the baseline (post-intervention), and 36 weeks after the baseline (follow-up) included clinical measurements, questionnaires of eating behavior (IES-1, TFEQ-R18, HTAS, ecSI 2.0, REBS), diet quality (IDQ), alcohol consumption (AUDIT-C), perceived stress (PSS), and 48-h dietary recall. Hierarchical linear modeling (Wald test) was used to analyze the differences in changes between groups.

**Results:**

Group x time interactions showed that the subcomponent of intuitive eating (IES-1), i.e., *Eating for physical rather than emotional reasons*, increased in both ACT-based groups (*p* = .019); the subcomponent of TFEQ-R18, i.e., *Uncontrolled eating*, decreased in the Face-to-face group (*p* = .020); the subcomponent of health and taste attitudes (HTAS), i.e., *Using food as a reward*, decreased in the Mobile group (*p* = .048); and both subcomponent of eating competence (ecSI 2.0), i.e., *Food acceptance* (p = .048), and two subcomponents of regulation of eating behavior (REBS), i.e., *Integrated* and *Identified regulation* (*p* = .003, *p* = .023, respectively), increased in the Face-to-face group. Baseline perceived stress did not moderate effects on these particular features of eating behavior from baseline to follow-up. No statistically significant effects were found for dietary measures.

**Conclusions:**

ACT-based interventions, delivered in group sessions or by mobile app, showed beneficial effects on reported eating behavior. Beneficial effects on eating behavior were, however, not accompanied by parallel changes in diet, which suggests that ACT-based interventions should include nutritional counseling if changes in diet are targeted.

**Trial registration:**

ClinicalTrials.gov (NCT01738256), registered 17 August, 2012.

**Electronic supplementary material:**

The online version of this article (10.1186/s12966-018-0654-8) contains supplementary material, which is available to authorized users.

## Background

Making long-term eating-related behavioral changes to promote health is difficult. We need to find ways to support people in making the changes [1]. Long-term changes seem to be associated, for example, with supporting individual’s autonomy and internal motivation [2]. Internal motivation for regulating eating means that one is engaged in health-related behavior for one’s own sake and free will and that one’s action is congruent with own values and goals [3].

Acceptance and commitment therapy (ACT) is one promising method in changing behavior towards a person’s own values and goals. ACT consists of six interrelated core processes: (1) clarification of own values, (2) commitment to act based on those values, (3) being in contact with the present moment (i.e., mindfulness), (4) having self as context (i.e., being aware of thoughts, feelings, etc. without attaching to them), (5) defusion (i.e., altering the way to interact with or relate to thoughts, feelings, etc.), and (6) acceptance [4]. Thus, ACT aims to strengthen positive psychological processes related to commitment, behavior change, mindfulness, and acceptance [4], which can be applied to promote healthy behavioral patterns [5]. Using ACT is supported by the promising results of ACT-based interventions on food cravings [6] and weight loss [7–13].

Furthermore, deficiency of one of the core processes of ACT, namely, mindfulness, during eating can lead to overeating or eating without physical hunger [14, 15]. Mindfulness training, instead, has reduced impulsive eating and binge eating in adults with overweight and obesity [16], has reduced energy intake in experimental settings [17, 18], and may thus increase consciousness of one’s eating behavior and its regulation.

One aim in mindful eating training is to increase awareness of bodily hunger and satiety cues and to eat according to them [19, 20]. The emphasis on bodily hunger and satiety cues is also included in two concepts of eating behavior: intuitive eating (i.e., having unconditional permission to eat whatever desired, and eating relying on hunger and satiety cues and not on emotions) [21, 22] and eating competence (i.e., having positive attitudes about eating and food, accepting and eating an ever-increasing variety of foods, eating according to internal hunger and satiety signals, and having skills and resources for managing daily meals) [23]. Both intuitive eating and eating competence have been associated with better diet quality [24, 25] and lower BMI [22, 26–28]. However, no previous ACT or mindfulness intervention studies have been targeted on eating competence, and those reporting effects on intuitive eating have had strong emphasis on intuitive eating approach in the intervention [29, 30]. Previous mindfulness-based interventions have been shown to decrease [31, 32] or to have no effect [9, 33] on emotional eating (i.e., eating based on negative emotions [34]). Thus, more research on the effects of ACT and mindfulness on eating behavior is needed.

There is also a need for new methods that are cost-effective to health care systems and easily accessible to the people who need support. New technology, such as mobile apps, have gained wide interest recently [35–38], and there is already some evidence that mobile apps can be effective in improving health-related behavior [39].

**Fig. 1:**
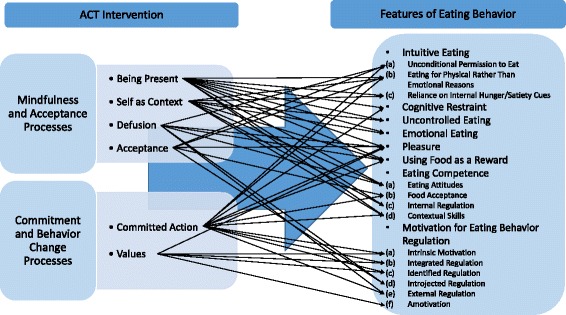
Theoretical model. The hypothesized effects of the core processes of Acceptance and Commitment Therapy (ACT) on the reported features of eating behavior

The aim of this study was to investigate the effects of ACT intervention delivered in two different ways, i.e., via face-to-face group sessions and via mobile app, on reported eating behavior and diet quality among adults with psychological distress and overweight or obesity. Because there is some evidence that human support enhances technology-based interventions’ effects [40–42], we hypothesized that the effects of an independently used mobile app ACT would be more modest than the effects of face-to-face ACT. The ACT intervention was not designed to specifically target eating behavior. However, several hypothesized effects are presented in Fig. 1. We found previously in this study population [43] that higher perceived stress is associated with unfavorable features of eating behavior: having less intuitive eating, eating competence and cognitive restraint, and more uncontrolled and emotional eating. Therefore, we also investigated whether baseline perceived stress moderates the effects of ACT on eating behavior.

## Methods

### Study design

The present study is a secondary analysis of the parallel-arm Elixir randomized controlled trial in which three different psychological interventions were studied [44]. The present study focuses on the effects of the two intervention arms based on ACT. See Lappalainen et al. [44] for the study protocol and participant flow chart.

The study participants were recruited by advertisements in local newspapers and screened for eligibility via telephone and on-line questionnaire from August 2012 until January 2013. The participants had to be 25–60 years old and have a self-reported body mass index (BMI) 27–34.9 kg/m^2^. The participants also had to be psychologically distressed (≥3/12 points from the General Health Questionnaire, GHQ-12 [45]) and have computer and Internet access. There were several exclusion criteria, such as diagnosed severe chronic illness including eating disorder, disabilities/illnesses affecting substantially physiological or mental health, pregnancy or breastfeeding within the past 6 months, psychotherapy or other psychological or mental treatment at least twice a month, and participation in other intervention studies during the present study. The multicenter study was conducted in three cities in Finland (Jyväskylä, Kuopio, and Helsinki) in two phases. The first phase started in autumn and the second phase in spring. The participants filled in electronic questionnaires, visited the local study center for clinical and biochemical measurements, and reported their food consumption in a 48-h dietary recall by telephone. Measurements were conducted before the intervention (baseline, study week 00), after the 8-week intensive intervention period (post-intervention, study week 10), and 36 weeks after baseline measurements (follow-up, study week 36). The measurements were collected from August 2012 until December 2013.

The sample size of the current study is based on the power calculation (for depression symptoms) of the Elixir randomized controlled trial [44], resulting a sample size of *n* = 80–85 per group.

### Ethics, consent and permissions

The study was approved by the ethics committee of the Central Finland Health Care District (reference number 7 U/2012) and was performed in accordance with the Declaration of Helsinki. The participants gave their written informed consent before participating. The study was registered with ClinicalTrials.gov with the identifier NCT01738256.

### Participants

Of the 254 individuals randomized to the Face-to-face, Mobile or Control groups, 219 participated in baseline measurements. At baseline, the participants’ (*n* = 219, 85% females) mean BMI was 31.3 kg/m^2^ (SD = 2.9), and their mean age was 49.5 years (SD = 7.4). The baseline demographic and clinical characteristics did not differ among the three study groups (Table 1). The number of participants at baseline, post-intervention, and follow-up were as follows: Face-to-face group—70, 62, and 60; Mobile group—78, 75, and 73; and Control group—71, 68, and 67, respectively. Thus, 89%, 96% and 96% of the Face-to-face, Mobile and Control group participants completed the post-intervention measurements, and 86%, 94% and 94% completed the follow-up measurements, respectively.

**Table 1:** Baseline demographic and clinical characteristics of each group

	Face-to-face	Mobile	Control	p^a^
Number of participants (n)	70	78	71	
Starting time of the study (n)				.642
Autumn	35	37	30	
Spring	35	41	41	
Study center (n)				.970
Jyväskylä	20	22	17	
Kuopio	22	25	23	
Helsinki	28	31	31	
Gender (n)				.670
Female	61	66	58	
Male	9	12	13	
Age (years)	50.3 ± 7.2	49.1 ± 7.7	49.2 ± 7.4	.575
Weight (kg)	86.1 ± 10.3	88.4 ± 10.4	88.3 ± 11.5	.342
BMI (kg/m^2^)	31.0 ± 3.1	31.6 ± 2.7	31.2 ± 2.8	.423
Psychological distress (GHQ-12 score)	7.2 ± 3.0	6.8 ± 2.8	7.4 ± 2.7	.408^b^
Perceived stress (PSS score)	25.8 ± 8.0	26.9 ± 7.8	26.9 ± 7.6	.597

Values are n / mean ± SD; Autumn = September – October 2012; Spring = January – February 2013; *BMI* body mass index, *GHQ-12* General Health Questionnaire-12, *PSS* Perceived Stress Scale

^a^*p*-value for differences between the study groups (Pearson chi-square for categorical variables and one-way ANOVA for continuous variables unless other noted)

^b^Non-parametric Kruskal-Wallis test

### Study groups

The Face-to-face and Mobile interventions were based on the same ACT program constructed by the same research group. Thus, only the delivery method of the intervention differed. The two interventions included the following main components: value clarification, acting according to own values, mindfulness skills, the observing self (e.g., observing thoughts without being caught up in them), and acceptance skills (e.g., making room for unpleasant feelings and urges allowing them to come and go). The main focus was on ACT skills but minor parts of mindful eating, relaxation, and everyday physical activity were also included. Mindful eating was the topic of one group session in the Face-to-face intervention group and of one section in the Mobile group’s app. The mindful eating component of the intervention consisted of learning to be present while eating; observe eating-related thoughts and feelings; observe and trust hunger and satiety cues; notice challenges for eating based on physical cues; be aware of the effects of not eating mindfully; recognize individual needs and feelings related to meal rhythm; and practicing mindful grocery shopping. Intervention did not include nutrition education. Only a hyperlink to a public nutritional web site was provided to the participants in intervention groups, which was to be utilized if the dietary changes were according to one’s values. See Lappalainen et al. [44] for a more detailed description of the intervention.

The Face-to-face group had six group sessions led by a psychologist during the 8-week intervention period. Each session took approximately 90 min, and each group consisted of 6–12 participants. The sessions included exercises, pair and group discussions, and homework for which the participants received a workbook.

The mobile group had one group session in which participants learned of the principles of ACT and received smartphones with the pre-installed Oiva mobile app [46]. The Oiva app contains 46 exercises in text and audio formats and introduction videos about the ACT skills. The user experience results of the app were positive [46]. The participants were free to choose exercises and videos in any order and to do them as many times as the participants wanted during the 8-week intervention period. The participants returned the smartphones during the post-intervention laboratory study visit. The participants’ usage of the mobile app is reported in detail by Mattila et al. [47].

Participants randomized to the Control group participated in all of the measurements and did not receive any intervention. After the follow-up measurements, the participants in the Control group had an opportunity to attend one group session in which principles of ACT were presented and to utilize the Internet-based lifestyle coaching program.

### Measures

#### Background characteristics

Weight and height were measured with calibrated instruments at each study center in the morning after a 12-h overnight fast [44]. BMI was calculated from the measured weight and height as kilograms per meters squared. The demographic information was collected using a questionnaire. The 12-item General Health Questionnaire, GHQ-12 [45], was used to screen the volunteers for psychological distress. The GHQ-12 has been found to be a valid screening tool for common mental health problems in the Finnish population [48]. The respondents were asked, considering the past few weeks, to answer questions such as “Have you recently felt capable of making decisions about things?” Bimodal scoring was used: “not at all” (0 points); “same as usual” (0); “rather more than usual” (1); and “much more than usual” (1), with the total sum score ranging from 0 to 12. Cronbach’s alpha was 0.72.

#### Outcome measures

##### Eating behavior

The Intuitive Eating Scale, IES [22], consists of 21 items with subcategories of intuitive eating: (a) Unconditional Permission to Eat (9 items, e.g., “If I am craving a certain food, I allow myself to have it.”), (b) Eating for Physical Rather Than Emotional Reasons (6 items, e.g., reversely scored “I find myself eating when I am bored, even when I’m not physically hungry.”), and (c) Reliance on Internal Hunger/Satiety Cues (6 items, e.g., “I trust my body to tell me when to eat.”). The statements are answered with a 5-point Likert scale. The scores are averaged; thus, the possible ranges of the IES total score and its subscales are 1–5. Cronbach’s alpha at baseline was 0.79 for the entire scale and 0.66, 0.84, and 0.77 for the subscales Unconditional Permission to Eat, Eating for Physical Rather Than Emotional Reasons, and Reliance on Internal Hunger/Satiety Cues, respectively. The questionnaire had been validated among college women in the USA [22].

The Three-Factor Eating Questionnaire, TFEQ-R18 [34], was used to measure (a) Cognitive Restraint (6 items, e.g., “I deliberately take small helpings as a means of controlling my weight.”), (b) Uncontrolled Eating (9 items, e.g., “Sometimes when I start eating, I just can’t seem to stop.”), and (c) Emotional Eating (3 items, e.g., “When I feel blue, I often overeat.”). The answers are given by 4-point Likert scale, except for one item, which is answered using an 8-point Likert scale. The possible range of the total scores was 0–100. Cronbach’s alphas were 0.71, 0.88, and 0.89 for the scales Cognitive Restraint, Uncontrolled Eating, and Emotional Eating, respectively. The Finnish version of the questionnaire had been validated in young, mostly normal weight, females and showed good structural validity [49].

Of the Health and Taste Attitude Scales, HTAS [50], subcategories (a) Pleasure (6 items, e.g., “When I eat, I concentrate on enjoying the taste of food.”) and (b) Using Food as a Reward (6 items, e.g., “I reward myself by buying something really tasty.”) were used. The statements were answered using a 7-point Likert scale. The scores were averaged; thus, the possible ranges were 1–7. Cronbach’s alphas were 0.71 and 0.81 for the subcategories Pleasure and Using Food as a Reward, respectively. The questionnaire developed in Finland had been validated among several general Finnish adult samples [50–52].

Eating competence was measured using a preliminary Finnish translation of ecSatter Inventory 2.0, ecSI 2.0 [28, 53, 54]. The definition of eating competence consisted of four components, which also constituted the 16-item questionnaire’s subcategories: (a) Eating Attitudes (5 items, e.g., “I am relaxed about eating.”), (b) Food Acceptance (3 items, e.g., “I experiment with new food and learn to like it.”), (c) Internal Regulation (3 items, e.g., “I eat as much as I am hungry for.”), and (d) Contextual Skills (5 items, e.g., “I generally plan for feeding myself. I don’t just grab food when I get hungry.”). The statements were answered: “always” (3 points), “often” (2), “sometimes” (1), “rarely” (0), or “never” (0). The possible ranges of the sum scores were as follows: Eating Competence total score, 0–48; Eating Attitudes and Contextual Skills, 0–15, and Food Acceptance and Internal Regulation, 0–9. Cronbach’s alpha was 0.76 for the whole scale and 0.58, 0.68, 0.59, and 0.75 for the subscales Eating Attitudes, Food Acceptance, Internal Regulation, and Contextual Skills, respectively. The questionnaire had been validated among mostly female, overweight and educated adult sample [26], low-income females [28, 53] and parents of preschool-age children [54] in the USA.

The motivation for eating behavior regulation was measured using the 24-item Regulation of Eating Behavior Scale, REBS [3]. The participants were asked to answer the question “Why are you regulating your eating behaviors?” with a 7-point scale ranging from “Does not correspond at all” (1) to “Corresponds exactly” (7). The scale measured autonomous forms of motivation: (a) Intrinsic motivation (e.g., “It is fun to create meals that are good for my health”), (b) Integrated regulation (e.g., “Eating healthy is an integral part of my life”), and (c) Identified regulation (e.g., “It is a good idea to try to regulate my eating behaviors”). In addition, there were controlled forms of motivation: (d) Introjected regulation (e.g., “I don’t want to be ashamed of how I look.”), (e) External regulation (e.g., “People around me nag me to do it.”), and (f) Amotivation (e.g., “I can’t really see what I’m getting out of it.”). Each category (a–f) included four items. The scores were averaged; thus, the possible ranges were 1–7. Cronbach’s alphas were 0.86, 0.89, 0.75, 0.60, 0.89, and 0.71 for a, b, c, d, e, and f, respectively. The questionnaire had been validated among female university students in Canada [3]. The Finnish version used in this study had been pilot-tested among a general adult sample (*n* = 37).

##### Food consumption and nutrient intake

A concise measure of food consumption, the Index of Diet Quality (IDQ) [55], consisted of 18 questions about frequency, portion size, and/or type of certain foods and drinks consumed during the previous month to evaluate adherence to Nordic and Finnish nutrition recommendations. The questions involved whole-grain products, fat-containing foods, liquid dairy products, vegetables, fruits and berries, sugary products, and the regularity of meal pattern. The answers were scored as either reflecting health-promoting diet (1 point) or not (0 points). Part of the questions (regarding both frequency and portion of the food or drink) were combined for the scoring, and thus the possible IDQ total score was 0–15. Points below 10 indicated non-adherence, and points from 10 to 15 indicated adherence to the health-promoting diet [55]. In this study, answers that seemed possibly unrealistic or outliers (e.g., 27 slices of bread per day) were confirmed with the participant, and corrections were made when needed. Answers that remained unverified (*n* = 1 at baseline, *n* = 2 at post-intervention) were coded as missing. The IDQ had been developed and validated among Finnish healthy, mostly normal weight, adult females using a seven-day food record [55].

Alcohol consumption during the previous six months was measured using the Finnish version of the questionnaire Alcohol Use Disorders Identification Test Consumption, AUDIT-C [56]. This questionnaire had been shown to have strong correlation to alcohol consumption in a general Finnish population [57]. The questionnaire contained three questions regarding the frequency and amounts of alcohol usage. For the questions concerning the amount of drinks consumed, a list of typical Finnish serving sizes and their corresponding amounts as standard drinks (e.g., 33 cl bottle of beer is one drink) were provided. The responses were scored from 0 to 4 and summed, and the possible total score was from 0 to 12. Cronbach’s alpha was 0.66.

The 48-h dietary recall was conducted to collect information on nutrient intake. The participants were asked to describe all of the foods and drinks consumed during the previous full 48 h (beginning at midnight and ending at midnight over two consecutive 24 h periods). The interview was conducted by trained nutritionists by telephone at a pre-scheduled time. The participants were told that the interview considered diet, but anything regarding 48-h recall was not mentioned beforehand. An electronic picture book [58] was used to help to describe portion sizes. The interviews were performed from Tuesday to Friday. The nutrient intake was calculated using AivoDiet software version 2.0.2.2 (Aivo Ltd., Turku, Finland) and the Fineli® Finnish Food Composition Database (National Institute for Health and Welfare, Nutrition Unit, Helsinki, Finland). The interview protocol of the 48-h dietary recall was created based on the face-to-face 48-h dietary recall conducted in the national FINDIET 2012 survey [59]. The 48-h dietary recall protocol of the Elixir study was designed by the three nutritionists who also conducted the interviews. The participants were encouraged to be truthful in the 48-h dietary recall and were told that the interviewer would not assess or comment on their eating and drinking or give any dietary counseling. The foods and beverages consumed during the 48 h were repeated at the end, and the interviewer encouraged the participant to make additions or modifications while repeating the course of the days’ events.

#### Moderator

##### Perceived stress

The Perceived Stress Scale, PSS [60], is a 14-item measure for assessing the degree to which a person perceives life as stressful. The questionnaire has demonstrated acceptable psychometric properties worldwide [61]. Questions concern how often a person has experienced certain feelings and thoughts during the previous month, e.g., “In the last month, how often have you found that you could not cope with all the things that you had to do?” The 5-point Likert scale from “never” (0) to “very often” (4) is summed for the total score (possible range 0–56). Cronbach’s alpha was 0.88.

### Statistical methods

The statistical analyses were performed using IBM SPSS Statistics version 21 and Mplus version 7.3. Pearson chi-square test, one-way ANOVA, and the Kruskal-Wallis test were used to test whether baseline demographic and clinical characteristics differed between the study groups.

Hierarchical linear modeling (HLM, Wald test) was used to analyze the group x time interaction, i.e., whether the three study groups changed differently between the measured time points (study weeks 00, 10, and 36). If there was a difference, post hoc tests were conducted to determine between the three study groups whether the difference was during the intensive intervention period (from study week 00 to 10) or after the intensive intervention period (from study week 10 to 36). HLM accounts for missing values at random (MAR) and includes all of the available data. The parameters were estimated using the full-information maximum likelihood method (MLR estimation in Mplus). The analyses were adjusted for study center and starting time of the study. *Emotional eating*, *External regulation*, and intake of *monounsaturated fat (E%)* differed significantly between the groups at baseline, and these analyses were conducted also adjusting for the baseline value. Exact *p*-values of Wald tests are shown in Table 2 and Additional file 1, whereas statistically significant *p*-values of the post hoc analyses are presented in the text.

**Table 2:** The effects of ACT-based Face-to-face and Mobile interventions on eating behavior

	Face-to-face				Mobile				Control				p^a^	d^b^
	0 wk	10 wk	36 wk	d^c^	0 wk	10 wk	36 wk	d^c^	0 wk	10 wk	36 wk	d^c^		
IES total score	2.9 ± 0.4	3.0 ± 0.5	3.1 ± 0.4	0.45	2.9 ± 0.5	3.0 ± 0.4	3.1 ± 0.5	0.29	3.0 ± 0.5	3.0 ± 0.5	3.0 ± 0.5	0.16	.090	0.27 0.13
Unconditional Permission to Eat	3.0 ± 0.5	3.0 ± 0.5	3.1 ± 0.5	0.10	3.1 ± 0.6	3.1 ± 0.6	3.1 ± 0.7	− 0.05	3.1 ± 0.6	3.0 ± 0.7	3.1 ± 0.6	− 0.01	.277	0.11 − 0.05
Eating for Physical Rather Than Emotional Reasons	2.4 ± 0.8	2.6 ± 0.7	2.8 ± 0.8	0.50	2.4 ± 0.8	2.6 ± 0.8	2.7 ± 0.8	0.44	2.6 ± 0.9	2.6 ± 0.8	2.7 ± 0.8	0.10	**.019**	0.40 0.33
Reliance on Internal Hunger/Satiety Cues	3.2 ± 0.6	3.3 ± 0.7	3.4 ± 0.6	0.36	3.2 ± 0.7	3.3 ± 0.6	3.4 ± 0.6	0.29	3.2 ± 0.7	3.2 ± 0.6	3.4 ± 0.6	0.27	.967	0.04 − 0.01
TFEQ-R18														
Cognitive Restraint	43.1 ± 16.6	49.4 ± 14.4	51.5 ± 17.0	0.47	45.2 ± 16.2	47.6 ± 17.6	48.7 ± 15.3	0.26	45.8 ± 15.3	48.4 ± 15.3	47.7 ± 16.2	0.11	.252	0.37 0.15
Uncontrolled Eating	49.3 ± 18.3	44.7 ± 20.1	39.5 ± 20.5	− 0.46	49.4 ± 20.1	44.6 ± 19.2	43.9 ± 20.3	− 0.30	50.2 ± 20.9	48.4 ± 21.0	47.7 ± 19.0	− 0.11	**.020**	− 0.34 − 0.20
Emotional Eating	64.9 ± 25.3	57.3 ± 24.6	54.6 ± 25.6	− 0.40	62.4 ± 27.5	56.3 ± 26.0	52.8 ± 25.8	− 0.36	55.9 ± 27.9	54.4 ± 28.9	53.8 ± 25.1	− 0.08	.083^d^	− 0.31 − 0.27
HTAS														
Pleasure	4.7 ± 0.9	4.8 ± 0.8	4.8 ± 0.9	0.16	4.9 ± 1.0	4.8 ± 0.9	4.7 ± 1.0	− 0.16	4.7 ± 1.0	4.7 ± 1.1	4.8 ± 1.0	0.15	.066	− 0.01 − 0.30
Using Food as a Reward	4.3 ± 1.2	4.2 ± 1.2	4.0 ± 1.2	− 0.21	4.6 ± 1.1	4.2 ± 1.2	4.1 ± 1.2	− 0.39	4.3 ± 1.2	4.2 ± 1.1	4.2 ± 1.2	− 0.10	**.048**	−0.11 − 0.29
ecSI 2.0 total score	26.2 ± 6.0	26.6 ± 6.3	28.1 ± 6.6	0.22	26.3 ± 5.7	26.8 ± 6.2	26.2 ± 6.2	− 0.04	25.8 ± 6.4	25.8 ± 5.9	26.5 ± 6.4	0.07	.164	0.14 −0.11
Eating Attitudes	10.0 ± 2.1	9.7 ± 2.1	10.0 ± 2.5	−0.03	9.7 ± 2.2	9.8 ± 1.9	9.4 ± 2.4	−0.12	9.7 ± 2.6	9.5 ± 2.6	9.7 ± 2.2	−0.03	.144	0.00 −0.09
Food Acceptance	4.9 ± 2.0	4.9 ± 1.9	5.5 ± 1.6	0.25	5.2 ± 1.9	5.1 ± 2.0	5.0 ± 2.0	−0.09	4.9 ± 1.8	4.9 ± 1.9	4.8 ± 1.9	−0.04	**.048**	0.31 −0.06
Internal Regulation	4.7 ± 1.9	4.8 ± 1.8	5.1 ± 1.7	0.22	5.0 ± 1.6	5.1 ± 1.6	4.7 ± 1.8	−0.17	4.8 ± 1.8	5.0 ± 1.5	4.9 ± 1.8	0.02	.077	0.20 − 0.18
Contextual Skills	6.7 ± 3.1	7.3 ± 3.1	7.6 ± 3.1	0.24	6.5 ± 3.0	6.8 ± 3.0	7.1 ± 3.2	0.18	6.4 ± 3.1	6.4 ± 2.6	7.1 ± 2.9	0.20	.720	0.05 − 0.02
REBS														
Intrinsic motivation	5.1 ± 1.2	5.3 ± 1.2	5.4 ± 1.2	0.19	5.1 ± 1.3	5.2 ± 1.2	5.2 ± 1.4	0.05	4.9 ± 1.4	4.9 ± 1.3	5.0 ± 1.4	0.09	.831	0.09 −0.04
Integrated regulation	3.9 ± 1.3	4.5 ± 1.3	4.7 ± 1.4	0.55	4.3 ± 1.3	4.4 ± 1.1	4.3 ± 1.4	0.09	4.1 ± 1.3	4.0 ± 1.3	4.2 ± 1.4	0.12	**.003**	0.41 − 0.04
Identified regulation	5.8 ± 0.9	6.0 ± 0.8	6.0 ± 0.8	0.17	5.9 ± 0.9	5.7 ± 1.0	5.7 ± 0.9	− 0.20	5.7 ± 0.9	5.6 ± 1.1	5.7 ± 0.9	0.02	**.023**	0.15 −0.21
Introjected regulation	4.2 ± 1.1	4.0 ± 1.2	4.0 ± 1.2	−0.13	4.2 ± 1.2	4.0 ± 1.2	4.1 ± 1.3	−0.09	4.3 ± 1.2	4.1 ± 1.2	4.1 ± 1.3	−0.19	.955	0.08 0.10
External regulation	3.0 ± 1.6	3.0 ± 1.6	2.9 ± 1.8	0.04	3.5 ± 1.6	3.5 ± 1.5	3.3 ± 1.6	−0.11	3.7 ± 1.7	3.5 ± 1.8	3.6 ± 1.7	−0.04	.489^e^	0.10 −0.07
Amotivation	2.1 ± 1.0	1.8 ± 0.9	1.7 ± 0.8	−0.39	2.1 ± 1.0	2.1 ± 1.0	2.1 ± 1.0	−0.00	2.1 ± 1.0	2.2 ± 1.0	2.1 ± 1.1	−0.00	.059	−0.36 0.00

The values are unestimated means ± SD. *IES* Intuitive Eating Scale, *TFEQ-R18* The Three-Factor Eating Questionnaire-R18, *HTAS* Health and Taste Attitude Scales, *ecSI 2.0* preliminary Finnish translation of Satter Eating Competence Inventory 2.0, *REBS* Regulation of Eating Behavior Scale. Higher scores represent higher amount of the feature in all of the scales. There were missing values of one (*n* = 1) participant in the Mobile group at week 36 and of three participants (*n* = 3) in the Control group at weeks 10 and 36

^a^*p*-value for differences in changes between the three study groups using all measured time points (study weeks 00, 10, and 36) adjusted for study center and starting time using estimated parameters (hierarchical linear model, Wald test). Bold text indicates significant *p*-value < 0.05

^b^Cohen’s d from baseline to follow-up between the Face-to-face and Control groups (above) and between the Mobile and Control groups (below) using estimated parameters

^c^Cohen’s d from baseline to follow-up within the group using estimated parameters

^d^After adding the baseline value to the adjustments, *p* = 0.088

^e^After adding the baseline value to the adjustments, *p* = 0.569

Cohen’s d was calculated from baseline to follow-up (Δ 36 weeks) within- and corrected between-groups to estimate effect sizes using the estimated values. A within-group effect size of 0.5 is considered small, 0.8 medium, and 1.1 large, and a corrected between-group effect size of 0.2 is considered small, 0.5 medium, and 0.8 large [62].

Baseline perceived stress was tested mean-centered as a moderator of the intervention effects on change in eating behavior from baseline to follow-up (Δ 36 weeks). Each outcome variable was tested separately in a single, saturated, moderation model in which the intervention groups were compared separately to the Control group using Mplus software. Maximum Likelihood (MLR) estimation was used.

## Results

### Treatment adherence

Of the data included in the analyses, most of the participants in the Face-to-face group attended either all six group sessions (*n* = 16, 23%) or five group sessions (*n* = 31, 44%). One participant did not attend any group sessions (*n* = 1, 1%) or attended only one (*n* = 1, 1%) or two group sessions (*n* = 1, 1%). The participants attended on average 4.7 group sessions. In the Mobile group, the median number of usage sessions of the mobile app was 21 (range 4–91, interquartile range IQR 11–33), according to the usage log files of the smartphones. The median number of usage days was 15 (range 4–59, IQR 8–23). The median total duration of use was 274 min (range 43–2001, IQR 181–421).

### Intervention effects on reported eating behavior

**Fig. 2:**
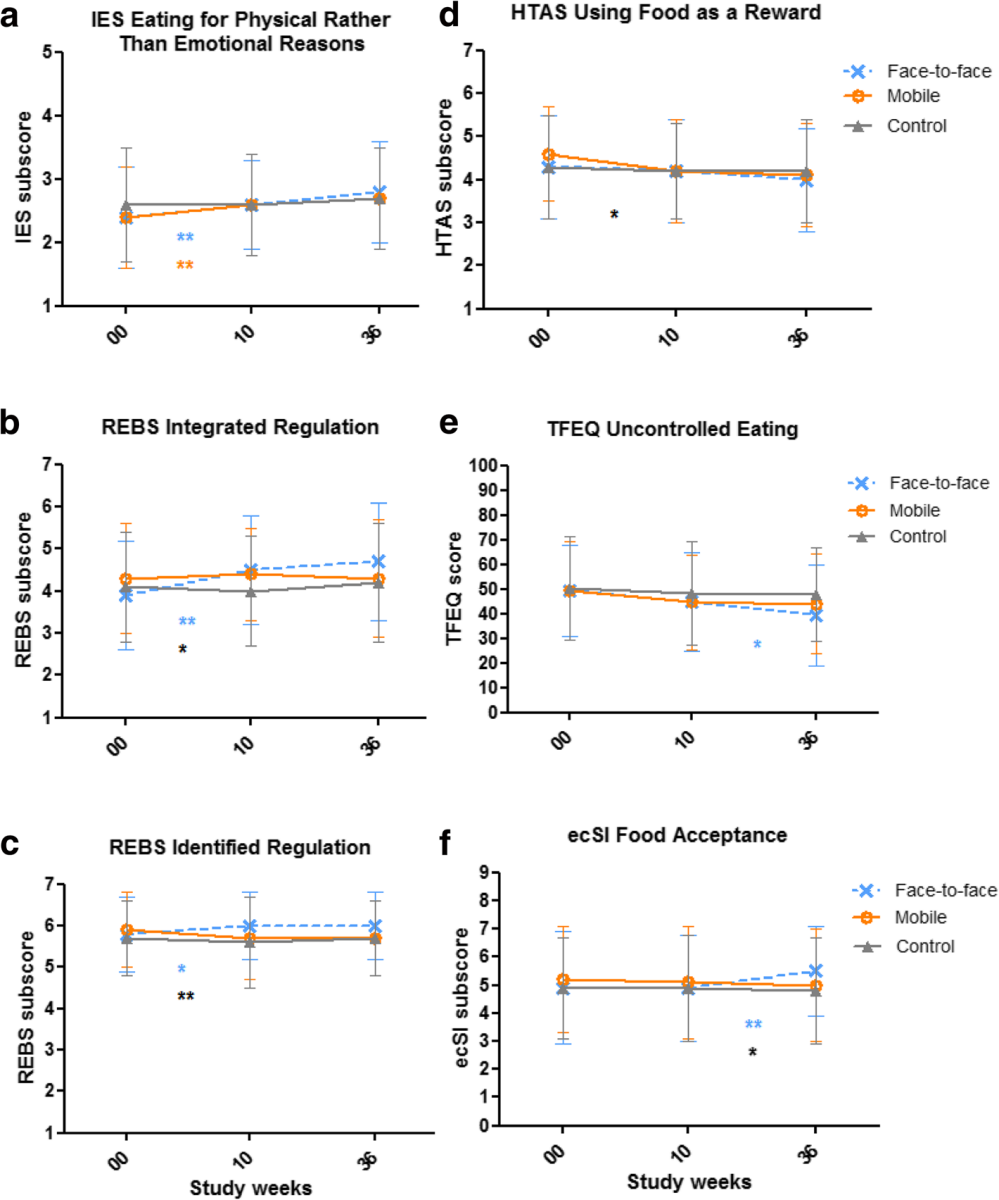
The statistically significant intervention effects. The measurements were conducted before the intervention (baseline, study week 00), after the 8-week intervention period (study week 10), and 36 weeks after the baseline measurements (study week 36). Face-to-face = Acceptance and commitment therapy (ACT)-based intervention, six group sessions led by a psychologist; Mobile = ACT-based intervention, one group session and mobile app; Control = only the measurements. The values are unestimated means ± SD. * *p* < 0.05, ** *p* < 0.01 adjusted for study center and starting time of the study. A blue asterisk (*) represents a difference between the Face-to-face group and Control, an orange asterisk (*) represents a difference between the Mobile group and Control, and a black asterisk (*) represents a difference between the Face-to-face and Mobile groups. IES = Intuitive Eating Scale; REBS = Regulation of Eating Behavior Scale; HTAS = Health and Taste Attitude Scales; TFEQ = The Three-Factor Eating Questionnaire-R18; ecSI = preliminary Finnish translation of Satter Eating Competence Inventory 2.0. Higher scores represent higher amount of the feature in all of the scales

Group x time interactions were found among the three study groups during the entire study period (study weeks 00, 10, and 36) in the following subcomponents: the subcomponent of intuitive eating (IES), i.e., *Eating for physical rather than emotional reasons*; the subcomponent of TFEQ-R18, i.e., *Uncontrolled eating*; the subcomponent of health and taste attitudes (HTAS), i.e., *Using food as a reward*; the subcomponent of eating competence (ecSI 2.0), i.e., *Food acceptance*; and two subcomponents of regulation of eating behavior (REBS), i.e., *Integrated* and *Identified regulation*, with small or small-to-medium effect sizes (*p* < 0.050) (Table 2). These differences are presented in more detail in Fig. 2 and in the following.

#### Changes from baseline to post-intervention (study weeks 00–10)

There were improvements in the subcomponents of intuitive eating (IES), regulation of eating behavior (REBS), and health and taste attitudes (HTAS) (Fig. 2a–d). *Eating for physical rather than emotional reasons* increased in both the Face-to-face and Mobile groups compared to the Control group (*p* = 0.007 and *p* = 0.006, respectively). *Integrated regulation* increased in the Face-to-face group compared to both the Control group and Mobile group (*p* = 0.001 and *p* = 0.027, respectively). Similarly, *Identified regulation* increased in the Face-to-face group compared to both the Control group and Mobile group (*p* = 0.033 and *p* = 0.004, respectively). *Using food as a reward* decreased in the Mobile group compared to the Face-to-face group (*p* = 0.027).

#### Changes from post-intervention to follow-up (study weeks 10–36)

There were improvements in the subcomponents of TFEQ-R18 and eating competence (ecSI 2.0) (Fig. 2e, f). *Uncontrolled eating* decreased in the Face-to-face group compared to the Control group (*p* = 0.014). *Food acceptance* increased in the Face-to-face group compared to both the Control group and Mobile group (*p* = 0.007 and *p* = 0.011, respectively).

#### Moderating effect of perceived stress

Baseline perceived stress did not moderate effects on the abovementioned features of eating behavior from baseline to follow-up (Additional file 2).

### Intervention effects on reported diet quality

There were no statistically significant differences in the changes in diet quality between the groups. The mean values, Cohen’s d, and *p*-values for the differences in changes between the three groups are presented in Additional file 1.

## Discussion

This study investigated the effects of ACT interventions that were delivered in group sessions or by mobile app on reported eating behavior and diet quality among adults with psychological distress and overweight or obesity. The ACT-based interventions showed beneficial effects on eating behavior with no parallel changes in diet. Our results suggest that ACT was able to change the reasons for eating from emotional or environmental triggers towards hunger and satiety cues, increase the acceptance of a variety of foods, and help the individual to perceive healthy eating more consistently with his or her own values and goals. The results are consistent with the ACT theory and related to all of the core processes of ACT. The effects were more pronounced in the Face-to-face group than in the Mobile group, although both showed positive changes. A subcomponent of *Intuitive eating*, *Eating for physical rather than emotional reasons,* increased in both ACT groups, and *Using food as a reward* decreased in the Mobile ACT-group during the intervention. Furthermore, internal (*Integrated* and *Identified*) motivation for regulating eating behavior increased in the Face-to-face ACT-group during the intervention. *Uncontrolled eating* decreased and *Food acceptance* increased in the Face-to-face ACT-group during the follow-up. The baseline perceived stress did not moderate the intervention effects on changes in these features of eating behavior from baseline to follow-up.

In the previous ACT or mindfulness intervention studies, effects have been shown on all subcomponents of *Intuitive eating* [29, 30], whereas in the present study, only the scores of subcomponent *Eating for physical rather than emotional reasons* increased. This result may be explained by different intervention contents because in previous studies, intuitive eating was included in all of the intervention sessions [29, 30], whereas our intervention consisted of general ACT with minor mindful eating component and no other eating-specific content. Our previous findings show that weight-related psychological flexibility seems to particularly mediate the effects of ACT on intuitive eating [63].

Although the ACT intervention increased *Eating for physical rather than emotional reasons* compared to the control, *Emotional eating* (measured by TFEQ-R18) did not change at a statistically significant level. The lack of an intervention effect compared to the control is in line with previous studies [9, 33]. However, our data showed a trend for a decrease in *Emotional eating* during the study period in both intervention groups, with small between-group effect sizes compared to the Control group. In line with this, decreased emotional eating compared to waitlist [31] and treatment as usual [13] have been reported. Considering the trend for decreased *Emotional eating*, the increased *Eating for physical rather than emotional reasons*, and the decrease in *Using food as a reward,* our results suggest that ACT can decrease eating for emotional reasons.

The effect of ACT on eating competence has not been studied previously. The ACT intervention in the present study with minor mindful eating component did not have an effect on the total score or subscales *Eating attitudes, Internal regulation,* or *Contextual skills.* Nevertheless, the *Food acceptance* subscale (e.g., “I experiment with new food and learn to like it.”) increased in the Face-to-face group compared to other study groups after the intensive intervention period, which suggests that the participants may have focused on learning general acceptance skills during the intensive intervention period and applied them to eating behavior later during the follow-up.

To the best of our knowledge, there are no previous studies on the effects of ACT on forms of motivation for eating behavior regulation. *Integrated* and *Identified regulation* (e.g., “Eating healthy is an integral part of my life”, “It is a good idea to try to regulate my eating behaviors”, respectively) increased in the Face-to-face group. This result is in line with the theory of ACT because *Integrated* and *Identified regulation* of behavior include acting consistently with one’s values [3]. Our results indicate that ACT can increase eating behavior based on personal values which in turn has predicted making healthier choices in the long term [3]. However, parallel changes in diet were not observed in our study.

Although the ACT had effects on reported eating behavior that have been associated with health-beneficial dietary intake, no effects were found on the index of diet quality, alcohol consumption or energy nutrient intake compared to the control. The lack of intervention effects on dietary measures may be due to several reasons. First, the ACT intervention did not include nutrition education, and only a hyperlink to a public nutritional web site was provided. Previous mindfulness-based interventions without strong or any emphasis on diet or eating have showed similar results [32, 64], whereas mindfulness-based interventions including also dietary information have shown improvement in diet [33, 65, 66]. Second, because ACT concentrates on psychological processes and overall behavioral change, the primary focus of the participants may not have been on dietary changes, and therefore these changes may have needed more time to occur. Furthermore, the scores of the index of diet quality indicate that, in general, the participants’ diet was health-promoting [55] already at the baseline, and thus there was no room for drastic changes. In the future, it would be interesting to study whether the changes in eating behavior mediate changes in dietary intake in the long term.

We found previously in the current study population that perceived stress was associated with several features of unbeneficial eating behavior reflecting less intuitive eating, less eating competence and less cognitive restraint, and more uncontrolled and emotional-based eating [43]. Of those features, ACT intervention improved three, namely, *Eating for physical rather than emotional reasons*, *Uncontrolled eating*, and *Using food as a reward*. More importantly, according to the moderation analyses, intervention effects on these features of eating behavior occurred regardless of the baseline perceived stress level.

ACT is usually studied delivered in group sessions, and this is the first time that its effects on reported eating behavior and diet quality delivered via mobile app have also been studied. Mobile-based solutions are seen as promising because they may save time and costs in health care and be easily accessible to patients [35–38]. The way that the two ACT interventions were delivered in our study seemed to impact eating behavior somewhat differently. The impact of the Face-to-face intervention seemed to be larger than what was observed in the Mobile intervention. In addition, all of the effects in the Mobile group occurred during the intensive intervention period, which suggested that there was an effect when the app was in active use [47]. The usage of mobile app was completely on the participant’s own responsibility, willingness, and remembrance. Thus, it may be possible that although the content of the ACT interventions were similar, the participants may have applied them differently because participating in the group sessions demanded intensive attention to the intervention contents. Furthermore, technology alone may not be as effective as intervention including human interaction [67]. Although the median duration of the mobile app usage was rather high, four and a half hours, the participants in Face-to-face group were more exposed to the treatment (on average seven hours). It is noteworthy that the mobile app was well accepted, e.g., the minimum number of usage days was four, and the median was fifteen.

### Strengths and limitations

The present study is unique in several ways. First, the ACT intervention was delivered in two different ways: face-to-face in group sessions and individually via mobile app. Second, the study examined the effects of general ACT, which included minor mindful eating component but no nutrition education. Third, the effects of ACT on this wide variety of eating behavior and diet quality measures have not been reported previously. Fourth, the study population consisting of working-aged adults with psychological distress and overweight or obesity without serious medical conditions is unique compared to the study populations of previous studies. A large sample size and multicenter design, representing three areas in Finland, is also a strength of this study. The participants were likely to be interested in lifestyle changes because they had all enrolled in the Elixir lifestyle intervention study voluntarily and thus represented a possible target group of ACT group treatment or Oiva mobile app users.

There were also some limitations in terms of generalizability and methodology. The generalization of the study results may be limited because most of the participants were female, and due to the exclusion of, for example, individuals with severe chronic illness, the study population does not represent all treatment-seeking individuals of the community. Furthermore, although the internal consistency reliability was high in most of the scales, two subscales of the ecSatter Inventory had rather low Cronbach’s coefficient alphas (< 0.6), which may reflect the small number of items in the subscales [68] or suggest that these were not reliable measures to use in this population. In addition, although all of the questionnaires had been validated in their original language, all of the Finnish translations had not been validated, especially among adults with overweight or obesity. The 48-h dietary recall telephone interviews were conducted instead of using food records to diminish the burden on the participants [69]. This retrospective method that considered a rather long time period could also be regarded as a limitation in our study. The outcome depends on participants’ memory, although this limitation was addressed in our interview protocol. The validity of the 48-h recall has rarely been studied, and the results have been partly controversial [70–72]. However, compared to a single 24-h recall, a 48-h recall is found to be superior [71]. The 48-h dietary recalls were performed from Tuesday to Friday, so Fridays and Saturdays are missing from the dietary intake data, which may have influenced our results because energy intake typically increases on weekends [73, 74]. It is also important to notice that, at baseline, the participants were unaware beforehand about the pre-scheduled telephone interview’s content. However, at post-intervention and follow-up, the participants have quite likely guessed what the scheduled telephone interview would involve, and they have had the possibility to change their eating to be able to report it as more socially desirable (more healthy food items and less unhealthier food items). Another consideration is related to the possibly increased attention towards eating in intervention groups because of the ACT skills and its effect on reporting food intake or eating behavior more accurately at post-intervention and follow-up.

## Conclusions

ACT-based interventions delivered in the Face-to-face group sessions or by the Mobile app showed beneficial effects on several aspects of reported eating behavior and were most pronounced in the Face-to-face group. However, the current general ACT intervention including only a minor mindful eating component is not enough to promote dietary changes. Thus, to affect diet, adding nutritional counseling to this form of therapy is suggested. Further studies on the effects of ACT-based skills that specifically target diet quality are needed. The ACT-based intervention could be a useful approach for people with overweight or obesity and difficulties in eating behavior. It is important to determine which populations would benefit most from face-to-face and mobile app interventions because both interventions could also be used in health care settings.

## Additional files

Additional file 1: **Table S1.** The effects of ACT-based Face-to-face and Mobile interventions on diet quality. (PDF 26 kb)

Additional file 2: **Table S2.** Standardized estimates (standard error) and *p*-values for moderated intervention effects. (PDF 27 kb)

## Abbreviations

ACT: Acceptance and commitment therapy
AUDIT-C: Alcohol Use Disorders Identification Test Consumption questionnaire
BMI: Body mass index
E%: Percentage of energy
ecSI 2.0: preliminary Finnish translation of Satter Eating Competence Inventory 2.0
GHQ-12: 12-item General Health Questionnaire
HLM: Hierarchical linear modeling
HTAS: Health and Taste Attitude Scales
IDQ: Index of Diet Quality
IES-1: Intuitive Eating Scale
MAR: missing at random
PSS: Perceived Stress Scale
REBS: Regulation of Eating Behavior Scale
TFEQ-R18: The 18-item Three-Factor Eating Questionnaire
