# Supplementary material for: The effects of acceptance and commitment therapy on eating behavior and diet delivered through face-to-face contact and a mobile app: a randomized controlled trial
Source: Int J Behav Nutr Phys Act. 2018 Feb 27;15:22. doi: 10.1186/s12966-018-0654-8 (PMC5828146; doi:10.1186/s12966-018-0654-8)
Supplement: Supplementary file 1 — Table S1. The effects of ACT-based Face-to-face and Mobile interventions on diet quality. (PDF 26 kb) [file 12966_2018_654_MOESM1_ESM.pdf]

Additional file 1: Table S1. The effects of ACT-based Face-to-face and Mobile interventions on diet quality.

	Face-to-face				Mobile				Control				p[a]	d[b]
	0 wk	10 wk	36 wk	d[c]	0 wk	10 wk	36 wk	d[c]	0 wk	10 wk	36 wk	d[c]		
Diet quality and alcohol use														
IDQ score[d]	10.3 ± 2.1	10.8 ± 2.2	11.1 ± 2.2	0.25	10.5 ± 2.0	10.8 ± 2.4	10.8 ± 2.0	0.14	10.2 ± 2.1	10.4 ± 2.3	10.9 ± 1.8	0.33	.471	-0.05 -0.19
AUDIT-C score[e]	3.4 ± 2.2	2.7 ± 2.1	2.7 ± 2.0	-0.24	3.9 ± 2.2	3.4 ± 2.2	3.4 ± 2.4	-0.24	4.0 ± 2.5	3.9 ± 2.5	3.6 ± 2.4	-0.15	.147	-0.07 -0.07
Intake of energy nutrients[f]														
Energy (MJ)	8.3 ± 1.9	7.7 ± 2.2	7.5 ± 2.1	-0.36	7.9 ± 2.1	7.9 ± 2.5	7.9 ± 2.2	-0.01	8.5 ± 2.7	8.7 ± 2.5	8.2 ± 2.4	-0.11	.148	-0.19 0.10
Protein (E%)	17.3 ± 4.2	19.0 ± 6.9	19.0 ± 5.0	0.37	18.0 ± 3.7	18.5 ± 5.1	19.3 ± 6.6	0.28	17.9 ± 4.4	17.6 ± 4.4	17.2 ± 5.0	-0.13	.173	0.48 0.35
Carbohydrate (E%)	41.3 ± 8.1	41.2 ± 7.2	40.3 ± 7.0	-0.17	39.4 ± 8.2	39.8 ± 8.0	39.6 ± 7.9	0.02	41.5 ± 7.2	40.4 ± 8.2	40.7 ± 7.8	-0.11	.810	-0.06 0.12
Total fat (E%)	37.7 ± 8.5	35.5 ± 7.7	36.9 ± 6.9	-0.07	38.2 ± 8.1	37.5 ± 7.8	37.2 ± 6.5	-0.14	36.2 ± 7.6	37.9 ± 6.4	37.4 ± 7.3	0.14	.215	-0.20 -0.27
Safa (E%)	13.3 ± 4.0	12.6 ± 3.8	12.7 ± 2.9	-0.17	13.8 ± 3.7	13.8 ± 4.4	13.3 ± 3.6	-0.14	13.4 ± 3.9	13.8 ± 3.2	13.3 ± 3.2	-0.04	.686	-0.15 -0.09
Mufa (E%)	13.4 ± 3.8	12.2 ± 3.1	13.1 ± 3.4	-0.06	13.2 ± 3.1	13.0 ± 3.2	12.8 ± 2.7	-0.12	12.1 ± 3.3	12.9 ± 2.7	12.9 ± 3.3	0.23	.102[g]	-0.26 -0.35
Pufa (E%)	6.5 ± 2.1	6.0 ± 1.8	6.5 ± 1.8	0.04	6.5 ± 2.2	6.2 ± 2.1	6.3 ± 1.8	-0.06	6.2 ± 2.2	6.4 ± 2.3	6.5 ± 2.4	0.09	.530	-0.06 -0.15
Sucrose (E%)	9.3 ± 5.3	9.4 ± 4.6	9.5 ± 4.9	0.03	8.9 ± 4.0	8.0 ± 4.1	8.4 ± 3.8	-0.14	9.5 ± 5.6	9.4 ± 4.7	8.8 ± 4.3	-0.14	.411	0.18 0.01
Fiber (g)	22.3 ± 7.7	20.1 ± 6.7	20.3 ± 7.9	-0.31	21.5 ± 7.9	20.7 ± 7.9	20.7 ± 7.9	-0.11	24.6 ± 11.6	22.3 ± 8.6	22.3 ± 8.1	-0.24	.578	-0.07 0.11
Fiber (g/MJ)	2.7 ± 0.8	2.7 ± 0.9	2.7 ± 0.8	0.01	2.8 ± 0.9	2.8 ± 1.0	2.7 ± 0.9	-0.11	2.9 ± 1.1	2.6 ± 0.8	2.8 ± 1.0	-0.12	.171	0.13 0.01

Notes: The values are unestimated means ± SD. IDQ = Index of Diet Quality (possible score range 0 – 15); AUDIT-C = three alcohol consumption questions from the Alcohol Use Disorders Identification Test (possible score range 0 – 12); E% = percentage of energy; Safa = Saturated fat; Mufa = Monounsaturated fat; Pufa = Polyunsaturated fat.

[a] p-value for differences in changes between the three study groups using all measured time points (study weeks 00, 10, and 36) adjusted for study center and starting time using estimated parameters (hierarchical linear model, Wald test).

[b] Cohen's d from baseline to follow-up between the Face-to-face and Control groups (above) and between the Mobile and Control groups (below) using estimated parameters.

[c] Cohen's d from baseline to follow-up within the group using estimated parameters.

[d] n=61 in the Face-to-face group at week 10, and n=66 in the Control group at week 36.

[e] n=66 in the Control group at week 36.

[f] n=74 in the Mobile group at week 10.

[g] After adding the baseline value to the adjustments, p=0.207.
