# Supplementary material for: The effects of acceptance and commitment therapy on eating behavior and diet delivered through face-to-face contact and a mobile app: a randomized controlled trial
Source: Int J Behav Nutr Phys Act. 2018 Feb 27;15:22. doi: 10.1186/s12966-018-0654-8 (PMC5828146; doi:10.1186/s12966-018-0654-8)
Supplement: Supplementary file 2 — Table S2. Standardized estimates (standard error) and p-values for moderated intervention effects. (PDF 27 kb) [file 12966_2018_654_MOESM2_ESM.pdf]

Additional file 2: Table S2. Standardized estimates (standard error) and p-values for moderated intervention effects.

Moderator (baseline)	Outcome (from baseline to follow-up, Δ 36 weeks)					
PSS	IES<br>Eating for Physical Rather Than Emotional Reasons	TFEQ<br>Uncontrolled Eating	HTAS<br>Using Food as a Reward	ecSI<br>Food Acceptance	REBS<br>Integrated regulation	REBS<br>Identified regulation
Face-to-face	-0.03 (0.10)<br>.798	0.04 (0.09)<br>.659	0.04 (0.09)<br>.679	-0.18 (0.10)<br>.082	-0.09 (0.10)<br>.368	-0.14 (0.12)<br>.245
Mobile	-0.03 (0.10)<br>.778	0.06 (0.11)<br>.607	-0.10 (0.09)<br>.277	0.05 (0.10)<br>.623	0.01 (0.11)<br>.930	-0.09 (0.13)<br>.520

Notes: The ACT intervention groups (Face-to-face and Mobile) are compared separately to the Control group. PSS = Perceived Stress Scale; IES = Intuitive Eating Scale; TFEQ = The Three-Factor Eating Questionnaire-R18; HTAS = Health and Taste Attitude Scales; ecSI = preliminary Finnish translation of Satter Eating Competence Inventory 2.0; REBS = Regulation of Eating Behavior Scale.
